# LrHSP17.2 Plays an Important Role in Abiotic Stress Responses by Regulating ROS Scavenging and Stress-Related Genes in *Lilium regale*

**DOI:** 10.3390/plants13172416

**Published:** 2024-08-29

**Authors:** Shaokang Tang, Qin Ling, Qiqi Ma, Yuqing Cheng, Peng Mei, Yuan Miao, Yuanzhi Pan, Yin Jia, Mengxi Wu, Xue Yong, Beibei Jiang

**Affiliations:** 1 College of Landscape Architecture, Sichuan Agricultural University, Chengdu 611130, China; tangsk2000@163.com (S.T.); lqlq6707@163.com (Q.L.);; 2 College of Forestry, Sichuan Agricultural University, Chengdu 611130, China

**Keywords:** heat shock proteins, *Lilium regale*, abiotic stress, ROS, abscisic acid, melatonin

## Abstract

As an important part of heat shock response module, heat shock proteins (HSP) play an important role in plant defense response against heat stress; however, the involvement of the majority of the HSP family members against other abiotic stresses remains poorly understood. In the present study, *LrHSP17.2* was identified and its function against abiotic stress was analyzed. The expression level of *LrHSP17.2* was significantly induced by heat. Heterologous transgenes of *LrHSP17.2* showed that *LrHSP17.2* can increase the activity of catalase, peroxidase, superoxide dismutase to removes excess reactive oxygen species (ROS), maintain the stability of the membrane structure, and regulate genes related to antioxidant enzymes and defense under abiotic stress. In addition, *LrHSP17.2* could be regulated by exogenous abscisic acid and melatonin, and the related hormone synthesis genes of transgenic plants were significantly up-regulated under heat stress. Taken together, our results revealed that LrHSP17.2 is involved in regulating abiotic stress responses by regulating ROS scavenging and stress-related genes in *Lilium regale*.

## 1. Introduction

With the deterioration of the global environment, problems such as high temperature, drought, and land salinization emerge one after another. Continuous environmental stress affects the growth and development of plants, resulting in reduced crop yields and reduced ornamental value of plants. As one of the famous cut flowers, lily is an important ornamental horticultural plant, and the effects of abiotic stress on their growth and development cannot be ignored. *Lilium regale* is a native lily in China, wild in high-altitude mountainous areas with harsh environments, and has excellent resistance to high temperature, drought, salinity, etc. Therefore, an understanding of the stress response mechanisms of *Lilium regale* under abiotic stress is essential to improve the tolerance of lily.

Heat shock proteins (HSPs) are a class of stress proteins that are newly synthesized or increased in cells or organisms when they are induced by environmental stress conditions such as high temperature, drought, hypoxia, and heavy metals [1]. Although the research on plant material started late [2], significant progress has been made in recent years. Plant heat shock proteins are classified in different ways and can be divided into six categories based on their molecular weight: HSP110, HSP90, HSP70, HSP60, HSP40, and sHSPs [3]. Among them, the most abundant and widely distributed in plants is the small heat shock protein, and the plant sHSPs have a larger number than other eukaryotic sHSPs, and the number varies in different species. It plays an important role in maintaining the quality of proteins in cells. With the completion of more and more plant whole genome sequencing, comprehensive identification and analysis of the sHSPs gene family in different species is gradually being carried out, including Arabidopsis thaliana, *Oryza sativa*, and *Solanum lycopersicum* [4,5,6]. According to the analysis of subcellular localization, sequence homology and phylogenetic relationship, the sHSPs gene belonged to different subclasses, including cytoplasmic type, mitochondrial type, endoplasmic reticulum type, etc. [7]. The basic sequence of sHSPs includes a variable-length N-terminal region (average length 55 amino acids), a conserved C-terminal region, and a C-terminal extension region [8]. The conserved C-terminal region is made up of about 90 amino acids that form multiple β folds, which are called α-crystallin domain, is a sign that the sHSP family is evolutionarily conserved. This domain is a characteristic structure that distinguishes small heat shock proteins from other heat-induced small proteins and may play an important role in the oligomerization process and chaperone activity of small heat shock proteins [9].

Plants inevitably interact with the environment and encounter different types of biotic and abiotic stresses during their growth. Previous studies have shown that in many plants, sHSPs play an important role in improving plant heat tolerance [10,11,12,13]. In addition to high temperature or heat stress stress, other biotic and abiotic stresses can also induce the expression of sHSPs genes, such as low temperature, drought, salt stress, heavy metal ions, ultraviolet radiation, osmotic stress, anaerobic stress, pathogenic bacteria, etc. [14]. These results not only contribute to the formation of heat tolerance in organisms, but also play an important role in the process of plant adaptation to the environment [15,16].

However, studies on sHSPs are primarily focused on crops or model plants, such as *Oryza sativa*, *Triticum aestivumt*, and *Arabidopsis thaliana*, and little research has been carried out on lily. Here, we identify a C-II sHSPs in *Lilium regale*, *LrHSP17.2*, which was induced by HS and can be involved in multiple abiotic stresses. The thermotolerance of transgenic plants increased with the overexpression of *LrHSP17.2*. Further analysis indicated that *LrHSP17.2* is also involved in resistance to drought and salt. Furthermore, it is induced by exogenous hormones and involved in hormone synthesis pathways. The results of this study may help to reveal the biological function and mechanism of sHSPs in lily under abiotic stress and provide an important theoretical foundation for further perfecting the abiotic stress signal transduction network regulated by sHSPs.

## 2. Results

### 2.1. LrHSP17.2 Is a Heat-Inducible Heat Shock Protein Member of Lilium regale

**Figure 1:**
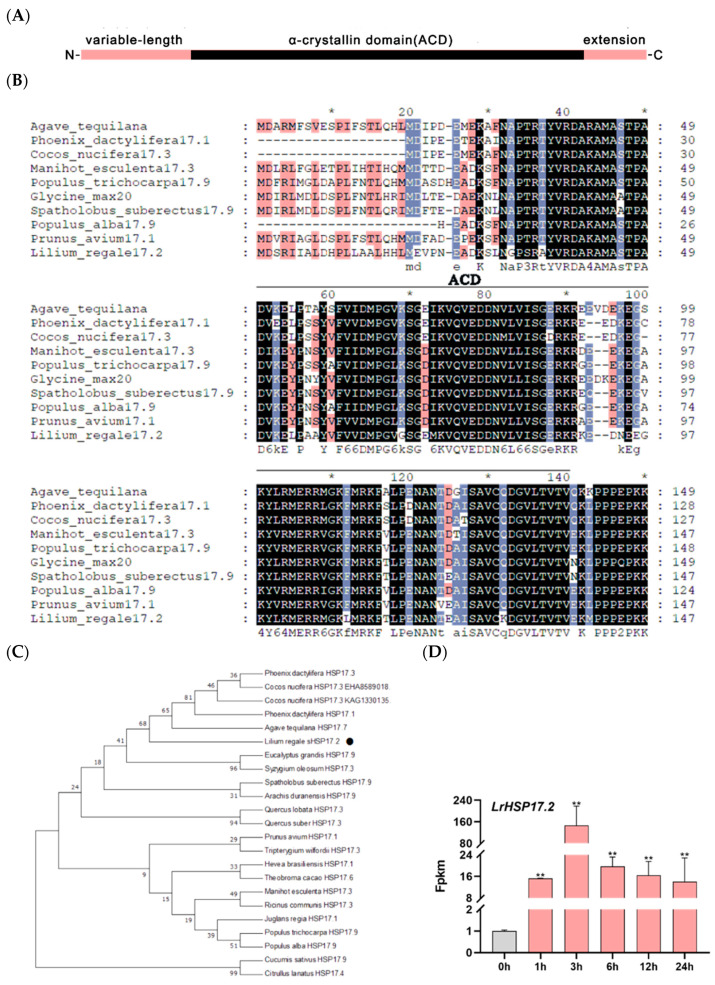
Amino acid sequence analysis and expression pattern analysis under heat stress at 45 °C. (**A**) Protein-conserved domains of *LrHSP17.2*; (**B**) protein sequence homology alignment of *LrHSP17.2*; * indicates that the amino acid sequence is conserved at this location; colors represent different homologies, black: homology = 100%, blue: homology ≥ 75%, red: homology ≥ 50%; (**C**) phylogenetic evolutionary tree of *LrHSP17.2*, *LrHSP17.2* has been marked with a black circle; (**D**) expression pattern of *LrHSP17.2* in the transcriptome under high temperature stress at 45 °C. *t*-test analysis of variance was employed to identify treatment means that differed statistically. Samples with different letters are significantly different: * *p* < 0.05, ** *p* < 0.01, unmarked means non-significant.

*LrHSP17.2* were isolated from leaves of *Lilium regale*. The full-length cDNA sequence of *LrHSP17.2* contains a 471-bp open reading frame (ORF) that encodes a 156-amino-acid protein. LrHSP17.2 contains a conserved ACD domain belonging to the HSPs p23-like family, indicating that the cloned gene is small heat shock protein of *Lilium regale* (Figure 1A). According to the naming rules of heat shock protein, the molecular weight of the protein encoded by it is 17.239 KDa, so it is named LrHSP17.2. The BLAST result revealed that the LrHSP17.2 with a high degree of similarity to AtHSP17.7, PdHSP17.1 and CnHSP17.3, etc., which were all cytoplasmic II type (C-II) small heat shock protein (Figure 1B). Then, the evolutionary relationship between LrHSP17.2 and other sHSPs in different plants was investigated, and the phylogenetic results indicated that closer relationships were established between LrHSP17.2 and noncereal monocot species (Figure 1C).

Next, we investigated whether *LrHSP17.2* expression in lily changes in response to heat stress treatments. The *LrHSP17.2* transcription levels were analyzed in transcriptome sequencing of plants that had been grown under normal growth condition and 45 °C heat at different time points (0, 1, 3, 6, 12, and 24 h). The results illustrated that *LrHSP17.2* expression was gradually induced by heat stress, and the expression level reached the highest at 3 h, and then decreased rapidly (Figure 1D). This suggested that *LrHSP17.2* is involved in a heat-responsive pathway, especially in the early stage of heat stress.

### 2.2. LrHSP17.2 Localizes to the Cytoplasm and Accumulates Near the Nucleus

**Figure 2:**
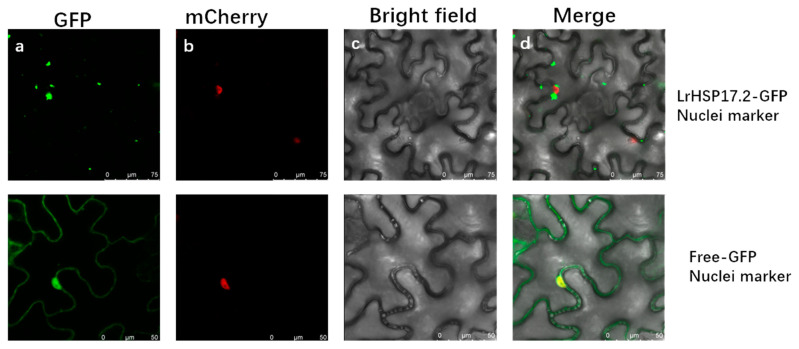
Subcellular localization of LrHSP17.2. It is distributed in aggregates in the cytoplasm surrounding the nucleus. (**a**–**d**) are GFP excitation images, mcherry excitation images, brightfield images, and merge images, respectively.

Fluorescence of the fusion protein LrHSP17.2-GFP was observed in the cytoplasm (Figure 2). In addition, most of them aggregate into particles, especially around the nucleus where larger particles form. These results once again confirmed that LrHSP17.2 is a cytoplasmic small heat shock protein, and since small heat shock proteins are molecular chaperones, they may work efficiently by aggregating multiple ones.

### 2.3. Overexpression of LrHSP17.2 Promote Root Growth at Seedling Stages

**Figure 3:**
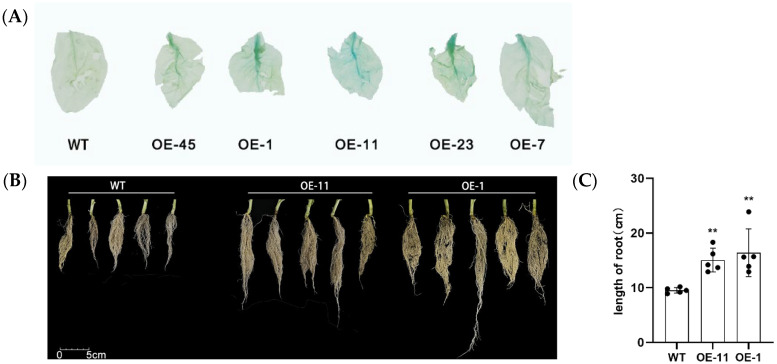
Identification of transgenic plants and growth differences. (**A**) GUS staining to identify transgenic plants; (**B**) comparison of plant root growth differences; (**C**) root length of different strains. Five independent experiments were performed, *t*-test analysis of variance was employed to identify treatment means that differed statistically. Samples with different letters are significantly different: * *p* < 0.05, ** *p* < 0.01, unmarked means non-significant.

To test the function of LrHSP17.2 in heat stress, LrHSP17.2 under the control of the 35s promoter was transformed into Nicotiana tabacum W38. Transgenic lines were screened by GUS staining assay and PCR. Two overexpression lines, OE-11 and OE-1, were chosen for heat stress phenotypic observation and physiological measurements with obvious staining (Figure 3A). What is novel is that root growth of the transgenic plants was significantly increased (Figure 3B,C). This is different from the usual growth defects associated with genetic modification.

### 2.4. Overexpression of LrHSP17.2 Enhances the Thermotolerance of Transgenic Plants by ROS Pathways

**Figure 4:**
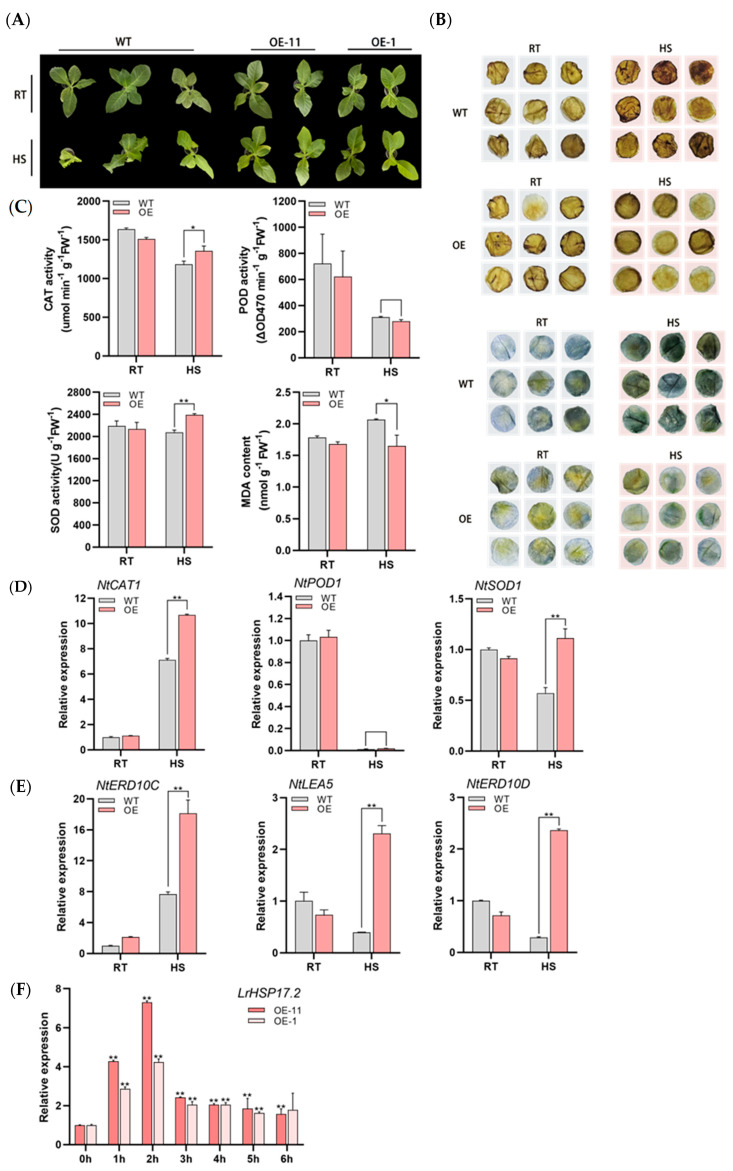
The thermotolerance was increased by overexpression of *LrHSP17.2*. (**A**) Phenotype of plants under 45 °C stress 3 h; (**B**) DAB and NBT staining of leaves to detect H_2_O_2_ and O^2−^ after HS (radius: 1 cm); (**C**) CAT, POD, and SOD activity and MDA content under heat stress; (**D**) expression of peroxidase genes under heat stress; (**E**) expression of stress defense genes under heat stress; (**F**) expression of *LrHSP17.2* in OE-11 and OE-1 under heat stress. *NtActin* was used as an internal standard. The physiological assay was determined by mixing samples of different OE strains. Three independent experiments were performed, each with three technical replicates, showing one experiment result. *t*-test analysis of variance was employed to identify treatment means that differed statistically. Samples with different letters are significantly different: * *p* < 0.05, ** *p* < 0.01, unmarked means non-significant.

Since LrHSP17.2 responds strongly to high temperature at 0–3 h, we chose HS at 45 °C for 3 h. Compared to the control, transgenic plants were significantly less vulnerable after HS (Figure 4A). The experiments measuring physiology and genes were determined by mixing samples of OE-11 and OE-1 strains to reduce individual error (In the following content, it will be uniformly referred to as OE). Under heat stress, reactive oxygen species will accumulate rapidly and cause harm to plants. Then, H_2_O_2_ content was detected by DAB staining in leaves of W38. At high temperature, the leaves of the control plants showed obvious dark red, while the transgenic plants did not show obvious staining (Figure 4B). In addition, superoxide anions (O^2−^) were also examined by NBT staining, and the result was the same as that of DAB staining—the staining of transgenic plants was lighter than control (Figure 4B). This indicated that less ROS accumulated in plants overexpressed under heat stress than in control plants. Therefore, LrHSP17.2 may improve the heat tolerance of plants by removing excess ROS.

To confirm the guess, some indexes of the protective enzyme system which mitigate and repair the damage initiated by ROS, were measured. Under HS conditions, the activities of SOD and CAT in transgenic plants were significantly higher than the control (Figure 4C), which was responsible for the lower contents of MDA and ROS in transgenic plants. Unexpectedly, the activity of POD did not seem to be related to transgenic of LrHSP17.2, and both decreased sharply under HS (Figure 4C). In addition, the expression levels of three antioxidant genes and three abiotic stress defense genes were measured using qRT PCR. The transcription levels of NtSOD1 and NtCAT1 in overexpressed plants were higher than control, while NtPOD1 showed no significant difference and decreased significantly, which was consistent with the enzyme activity measurements (Figure 4D).

A large number of studies have shown that NtERD10C, NtERD10D, and NtLEA5 play an important role in plant defense stress [17], and the transcription levels of these three genes are increased in LrHSP17.2 transgenic plants after heat stress, especially NtERD10C (Figure 4E). Interestingly, the response trend of LrHSP17.2 to heat in tobacco was different from that of lily, and its expression reached a peak at 2 h—which was obviously faster than that in lily—which may be related to the basic heat tolerance of plants (Figure 4F). These data indicate that LrHSP17.2 overexpressed plants can improve the heat tolerance of plants not only by increasing the expression of related antioxidant genes and enzyme activity to improve the ability to clear ROS but also by regulating the expression of defense-related genes.

### 2.5. Promoter Cloning and Analysis of LrHSP17.2

Via Tail-PCR, we obtained the upstream promoter sequence of *LrHSP17.2* with a length of 2147 bp. The sequence was analyzed using PlantCARE and NEW PLACE online promoter analysis sites, and a variety of hormone and transcription factor response elements were found (Table 1). Unexpectedly, the promoter of *LrHSP17.2* does not have the heat shock elements present in most heat shock proteins, but there are multiple heat-enhancing elements, which means that it is possible that *LrHSP17.2* is not directly regulated by heat shock transcription factors (HSFs). On one hand, ABA and methyl jasmonate (MeJA) usually act as regulatory hormones of abiotic stress, which can significantly improve plant resistance to abiotic stress. On the other hand, MYB and WRKY transcription factors have also been shown to be fully involved in abiotic stress in different plants. These results suggest that *LrHSP17.2* may be regulated by a variety of hormones and transcription factors and be involved in multiple abiotic stresses.

### 2.6. LrHSP17.2 Is Involved in Drought and Salt Stress Regulation in Transgenic Tobacco Plants

**Figure 5:**
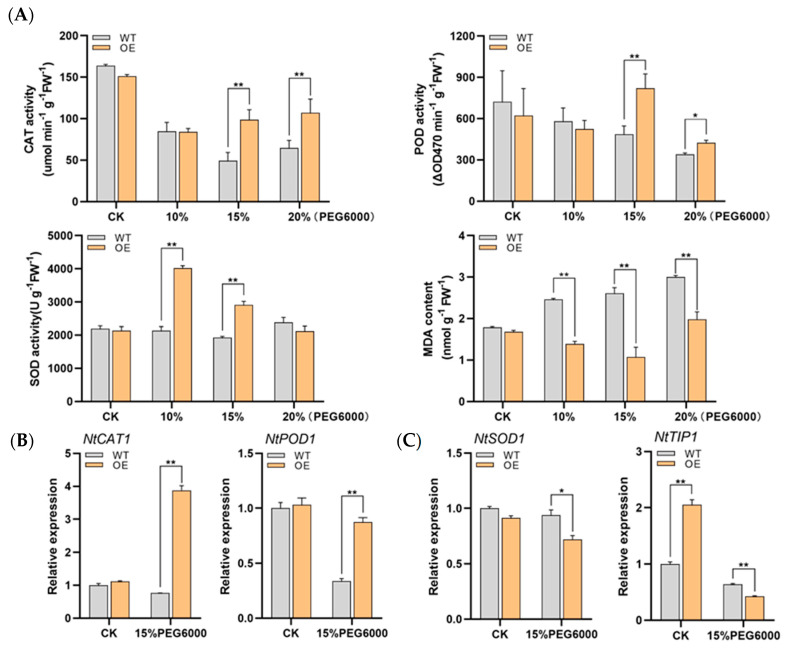

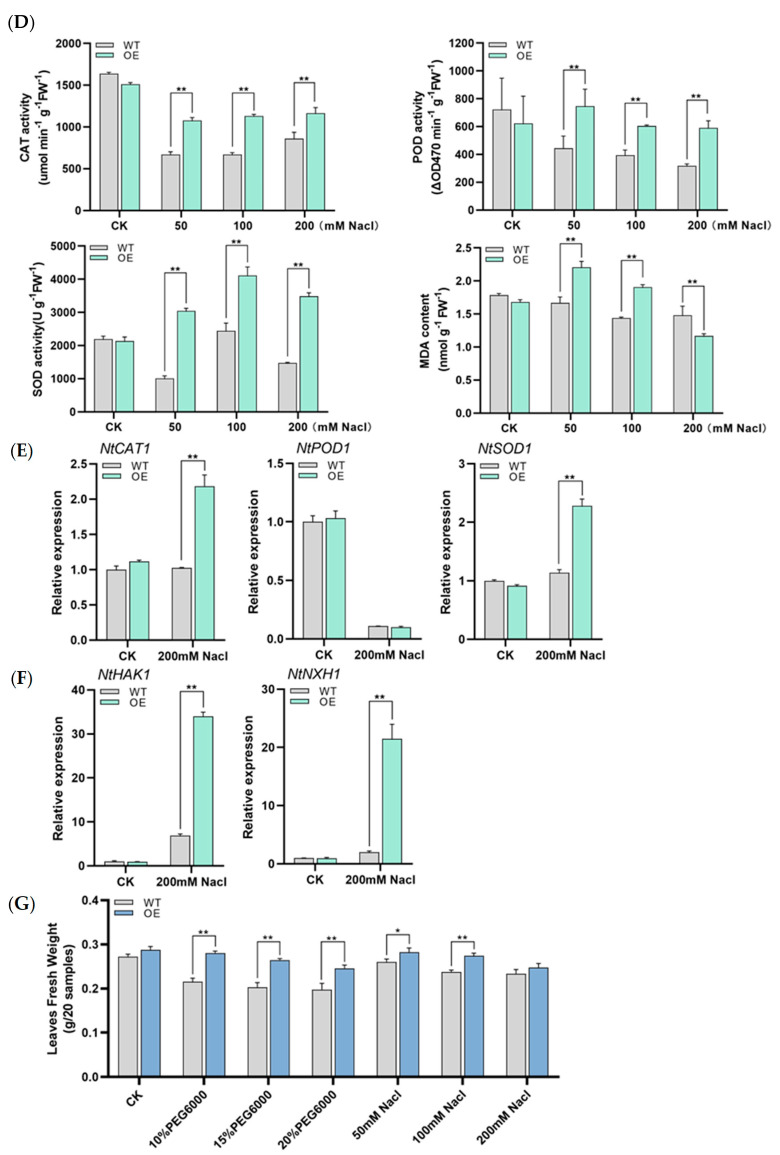
The tolerance of drought and salt stress was increased by overexpression of *LrHSP17.2* in tobacco. (**A**) CAT, POD, and SOD activity and MDA content under drought stress; (**B**) expression of peroxidase genes under drought stress; (**C**) expression of aquaporin genes under drought stress; (**D**) CAT, POD, and SOD activity and MDA content under drought stress; (**E**) expression of peroxidase genes under drought stress; (**F**) expression of ion channel protein genes under drought stress; (**G**) fresh leaf weight under drought and salt stress. The physiological assay was determined by mixing samples of different OE strains. NtActin was used as an internal standard. Three independent experiments were performed, each with three technical replicates, showing one experiment result. *t*-test analysis of variance was employed to identify treatment means that differed statistically. Samples with different letters are significantly different: * *p* < 0.05, ** *p* < 0.01, unmarked means non-significant.

Since sHSPs are speculated to be involved in multiple abiotic stresses and DRE elements are present on the promoter, the resistance of *LrHSP17.2* transgenic plants to drought and salt stress was investigated. Values of 10%, 15%, and 20% PEG6000 were used to simulate mild, moderate, and severe drought stress for 5 d; the control check (CK) was treated with H_2_O. Under mild drought conditions, only SOD activity increased significantly in transgenic plants (Figure 5A). With the increase of drought, the activities of POD enzyme and CAT enzyme in transgenic plants increased, especially in moderate drought (Figure 5A). The MDA content of transgenic plants was lower than that of control under the three drought conditions, indicating that *LrHSP17.2* played a role in the three drought stress conditions (Figure 5A). Under moderate drought, the expression levels of *NtPOD1* and *NtCAT1* in transgenic plants were significantly higher than those in the control, but there was decrease in the expression level of *NtSOD1* (Figure 5B). In the case of drought, the osmotic potential inside the plant is greater than outside the plant, and the plant will lose water. Therefore, the gene expression level of the *NtTIP*, an aquaporin gene, was examined [18]. Under normal conditions, the *NtTIP* expression level of transgenic plants was significantly higher than that of the control, and more aquaporins may be more conducive to water utilization. However, drought stress causes *NtTIP* to be significantly down-regulated in leaves of transgenic plants (Figure 5C), which may result in reduced membrane permeability to avoid water loss.

Similarly, NACL solutions of 50, 100, and 200 were used to simulate low, medium, and high salt stress for 5 d. Under the three concentrations of salt stress, the activities of CAT, POD, and SOD in transgenic plants were significantly higher than those in the control group (Figure 5D). However, MDA in transgenic plants decreased significantly only under high concentration of salt stress, indicating that under medium- and low-concentration salt stress, ROSs are not the main harmful substances (Figure 5D). Then, the expression levels of three antioxidase genes under high salt stress were examined. Similar to high temperature stress, the expression levels of *NtCAT1* and *NtSOD1* in transgenic plants except *NtPOD1* were significantly higher than in the control (Figure 5E). Excessive salt in soil can affect water transport and osmotic regulation, and ion channel proteins play an important role in the low Na^+^ state in plants. Therefore, the expression changes of two ion channel proteins [19,20], *NtNXH1* and *NtHAK1*, were investigated in *LrHSP17.2* transgenic plants under high concentration salt stress, and the results were significantly higher than t control (Figure 5F), indicating that *LrHSP17.2* can improve the ability of plants to transport Na^+^ and absorb K^+^, which maintained Na^+^ at low levels.

In addition, since both drought stress and salt stress can cause changes in plant osmotic pressure and water loss, we compared the water content of plant leaves after stress (Figure 5G). The results showed that the leaf water content of transgenic plants was significantly higher than that of control except high concentration salt stress, indicating that *LrHSP17.2* effectively prevented the water loss of plants under stress.

In summary, the results indicate that *LrHSP17.2* is involved in drought and salt stress regulation. It can effectively remove excess ROS and promote the synthesis of other stress-related proteins.

### 2.7. LrHSP17.2 Is Induced by ABA and MT and Participates in Their Synthetic Pathways

**Figure 6:**
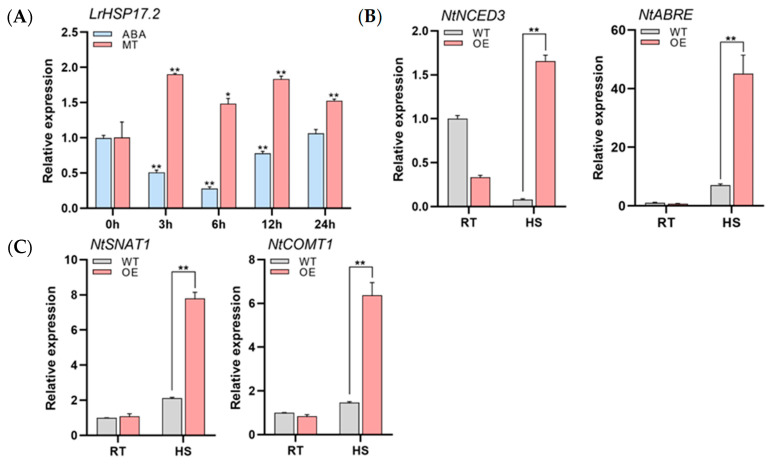
*LrHSP17.2* responses to exogenous ABA and MT and regulation of their synthetic genes. (**A**) The expression of *LrHSP17.2* in *Lilium regale* under exogenous ABA and MT treatments, *LrNADPH* used as an internal standard; (**B**) the expression of ABA synthesis gene under heat stress; (**C**) the expression of MT synthesis gene in tobacco under heat stress. *NtActin* was used as an internal standard. Three independent experiments were performed, each with three technical replicates, showing one experiment result. *t*-test analysis of variance was employed to identify treatment means that differed statistically. Samples with different letters are significantly different: * *p* < 0.05, ** *p* < 0.01, unmarked means non-significant.

Our previous work found that exogenous ABA and MT treatments can improve the heat resistance of *Lilium regale* (Supplementary Figure S1). The results also illustrated that at normal temperatures *LrHSP17.2* expression was significantly decreased under exogenous ABA and increased under exogenous MT in *Lilium regale* (Figure 6A). Hence, we determined the expression levels of *NtNCED3*, a gene associated with ABA biosynthesis, and *NtABRE*, a binding factor for ABA response elements [21]. Under heat stress, the expression levels of *NtNCED3* and *NtABRE* in transgenic tobacco plants were significantly higher than in the control (Figure 6B). So did two genes involved in MT synthesis [22,23] (Figure 6C), suggesting that *LrHSP17.2* possibly regulates the biosynthesis of ABA and MT to participate in the high-temperature signaling pathway, thereby improving the heat tolerance of plants. As to why ABA treatment reduces *LrHSP17.2* expression levels in *Lilium regale* at room temperature, it may be a feedback mechanism for plants to maintain ABA homeostasis, which requires later detection of hormone levels.

## 3. Discussion

Small heat shock protein, as members of the heat shock protein family, are known to participate in plant responses to abiotic stresses such as heat, cold, salt, and drought [24]. Their overexpression can significantly improve the stress tolerance of transgenic plants [25,26]. The functions of sHSPs have been widely and deeply studied in the eudicot model plant Arabidopsis and the crops rice, wheat, and maize, but their study in monocot ornamental plant has been less reported [16,27,28,29]. In this study, we identified and characterized a heat-inducible small heat shock protein from *Lilium regale*, LrHSP17.2, which had a typical ACD domain, was located in the cytoplasmic, and could participate in multiple abiotic stresses (Figure 1, Figure 2, Figure 3, Figure 4 and Figure 5).

Based on subcellular localization in cytoplasmic [30], together with phylogenetic analysis and peptide sequences, the homologous *LrHSP17.2* is considered to be a critical member of the C-II subtype family (Figure 1). As for function, sHSPs are not only closely related to plant development but also have the ability to resist stress. In addition, sHSPs is a kind of molecular chaperone which can prevent the accumulation of denatured proteins under stress conditions, effectively help protein folding and assembly, and alleviate external damage. In Arabidopsis, *AtHSP17.6* can improve the resistance to high temperature by increasing the activity of related antioxidant enzymes [26]. Additionally, *LrHSP17.2* transgenic plants significantly increased the activities of CAT, SOD and POD activity under stress, and decreased the content of MDA in vivo. In addition, *LrHSP17.2* transgenic plants also increased the expression of antioxidant enzyme genes and stress-related genes under stress. There may be differences in the trend of enzyme activity and the expression of synthetic genes, and this may be related to differences in response at the transcription level and protein level. This suggests that *LrHSP17.2* can alleviate external harm by removing excess ROS from plants and engaging in multiple defense pathways (Figure 4 and Figure 6).

In general, HSFs regulates HSP expression directly through the HSE element attached to the HSP gene promoter, which is a core module in heat stress response [31,32]. The promoter of *LrHSP17.2* contained many cis-elements involved in ABA- and dehydration-responsive pathways, but the conserved HSE was absent (Table 1), suggesting that expression of *LrHSP17.2* may be independent of HSFs. In Arabidopsis and tomato, sHSPs not only showed obvious stress expression during heat stress but also actively responded to other stresses, such as low temperature and drought [15,33,34]. In *Lilium regale*, *LrHSP17.2* participates in high temperature stress, drought, and salt stress. Due to the presence of low temperature and hypoxia response elements on its promoter, we speculate that *LrHSP17.2* may also participate in resistance to cold and flooding stress (Table 1).

When plants encounter adverse factors in the process of growth, they will improve their resistance through hormone regulation. As one of the five major hormones in plants, ABA plays an important role in plant response to stress. Exogenous ABA treatment can increase the synthesis and accumulation of proline and enhance the resistance to stress in rice [35,36]. In the biosynthesis pathway of abscisic acid, the high expression of key synthetic genes can increase the content of ABA in plants under stress, thus improving resistance. Overexpression of *AtNCED3* can significantly increase ABA content and drought resistance [37]. As a hormone with powerful physiological functions, melatonin has attracted much attention in recent years. Melatonin is a growth regulator and antioxidant that plays an important role in plant resistance to abiotic stress [38,39]. Pretreatment of tomato and wheat seedlings with 100 μmol/L melatonin can significantly reduce the accumulation of O^2−^ and H_2_O_2_ at high temperature and effectively relieve oxidative stress. In addition to ROS removal, melatonin treatment can also increase the chlorophyll content of cherry radish and protect the light and system [40]. In our previous studies, exogenous ABA and MT treatment also effectively improved the heat resistance of lily and increased the chlorophyll level, while *LrHSP17.2* was also regulated by these two hormones. In addition, genes related to ABA and MT synthesis in *LrHSP17.2* transgenic plants were significantly upregulated compared with controls under heat stress (Figure 6), suggesting that *LrHSP17.2* may be involved in hormone synthesis pathways.

**Figure 7:**
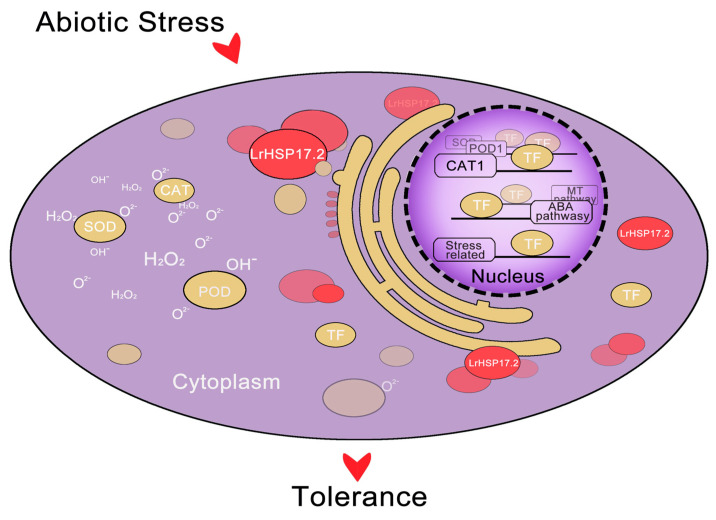
A proposed working model of LrHSP17.2 involved in resistance to abiotic stress. When *Lilium regale* suffers from abiotic stress, LrHSP17.2 is rapidly induced, and its protein can exert a chaperone function. This includes improving antioxidant enzyme activity and promoting the folding and formation of transcription factor proteins, thereby regulating hormone synthesis pathway genes, antioxidant enzyme genes, and stress-response genes.

According to the current results, we infer that *LrHSP17.2* enhance plant resistance by efficiently playing their molecular chaperone functions to help proteins associated with abiotic stress, especially transcription factors, fold and transform correctly under stress, and then regulate the expression of target genes (Figure 7). The transcription factors that interact with *LrHSP17.2* require further research.

## 4. Materials and Methods

### 4.1. Plant Materials and Growth Conditions

The *Lilium regale* used in this experiment was cultured on soil. Tobacco W38 used for genetic modification was cultured on MS, which is used in stress experiments, and tobacco is grown in soil. All plant materials were cultured in a growth room at 22 °C with a 16 h photoperiod, humidity 50–70%, and light intensity 10,000 Lux.

### 4.2. Molecular Cloning and Sequence Analysis of LrHSP17.2

Total RNA was extracted from the leaves of *Lilium regale* with an RNA extraction kit according to the manufacturer’s instructions (RC401 Vazyme, Nanjing, China). cDNA was bio-synthesized using a reverse transcription system (G891 Abm, Vancouver, BC, Canada). Primers for gene cloning are listed in Supplementary Table S1. Homology and conserved domains were analyzed via BLAST in NCBI. Phylogenetic evolutionary tree was constructed by MEGA11.

### 4.3. Subcellular Localization of LrHSP17.2

The ORF without the stop codon of LrHSP17.2 was inserted into a pCAMBIA2300-GFP vector to generate a fusion protein (LrHSP17.2-GFP). The primers used for vector construction are shown in Supplementary Table S1. The empty vector pCAMBIA2300-GFP was used as the control. The fluorescent protein carried by the nuclear mark is the mCherry, and the maximum absorption/emission peaks of mCherry are located at 587 nm and 610 nm, respectively. The plasmids pCAMBIA2300-*35s*-GFP and pCAMBIA2300-*35s*-*LrHSP17.2*-GFP were transformed into A. tumefaciens strain GV3101, and then, the bacterial solution was mixed 1:1 with nuclear marker infiltrated into *N. benthamiana*. A confocal laser-scanning microscope (LSM800, Zeiss, Jena, Germany) was used to observe the fluorescence.

### 4.4. Heat Stress Treatment of Lily

To detect gene expression levels under HS, one-month-old *Lilium regale* plants were exposed to 45 °C for 0, 1, 3, 6, 12, and 24 h. Heat treatment was applied in a temperature incubator for a Light/dark 12 h. Samples of leaves were harvested for transcriptome sequencing after heat treatment. Based on the Illumina technology sequencing platform, the transcriptome sequencing analysis was completed by using the method of double-terminal sequencing (Novogene, Beijing, China).

### 4.5. Gene Expression Analysis in Lily

Total RNA was extracted using the kit as described above. A HiScript II Kit with gDNA Eraser (R312 Vazyme, Nanjing, China) was used for cDNA biosynthesis with anmoligo dT primer. qRT-PCR was performed using a 20 μL reaction system. *LrGAPDH* was used as the endogenous gene. Three independent technical replicates were performed for each of three biological replicates. The relative expression level was calculated with the 2^–ΔΔCt^ method [41,42]. The primers for qRT-PCR are shown in Supplementary Table S1.

### 4.6. Isolation and Analysis of Promoter Sequences

The genomic DNA of *Lilium regale* was extracted using CTAB, following the manufacturer’s instructions. The promoter was isolated using hi-TAIL PCR. PLACE databases (http://www.dna.affrc.go.jp/PLACE/, accessed on 2 April 2023) and PlantCare (https://bioinformatics.psb.ugent.be/webtools/plantcare/html/, accessed on 3 April 2023) were used to analyze the cis-elements in the promoters.

### 4.7. Plant Transformation and Generation of Transgenic Lines

The model plant Nicotiana tabacum was used to verify the biological function of *LrHSP17.2* under stress. The bacterial solution of pCAMBIA1300-*35s*-*LrHSP17.2* was collected by centrifugation and resuspended in a Mgcl_2_ solution. Tobacco plant genetic transformation was performed according to the leaf disc method. Seedlings were used to extract RNA for RT-PCR identification of the transgenic lines and GUS staining.

### 4.8. Abiotic Stress Treatment of Tobacco

The harvested seeds were cultured and selected on soil until the T2 generation; then, T2 seedlings were used to treat. Tobacco at 5–8 leaf ages was used for heat, drought and salt stress. The heat stress was 45 °C for 3 h. Drought stress was simulated with 10%, 15%, and 20% PEG6000 for 5 days. The salt stress was treated with 50 mM, 100 mM, and 200 mM NaCl solution for 5 days. The treatment is treated by watering until liquid emerges from the bottom of the pot. Under drought and salt stress, light and temperature conditions are normal.

### 4.9. Physiological Assays

After these treatments, the phenotypes of the seedlings were recorded. Tobacco leaves were sampled with a 1 cm perforator for NBT and DAB staining and leaf water content determination. Then, the leaves were cut, sampled, and frozen in the −80 °C refrigerator for physiological measurements and gene expression analysis. The enzyme activities of CAT, SOD, and POD and the content of MDA were determined according to the kit protocol (G0105F, G0107F, G0101F Grace Biotechnology, Suzhou, China).

### 4.10. Exogenous Hormone Treatment

*Lilium regale* was sprayed with 30 mg/L ABA and 100 μmol/L MT. Controls were sprayed with the corresponding solvent or sterile ddH_2_O and treated with a spray until the liquid dripped off the leaves. After flash-freezing with liquid nitrogen, the material was stored in an environment of −80 °C.

### 4.11. Related Gene Expression in Transgenic Plants

Extract the RNA of the stressed tobacco and reverse transcribe to cDNA, which was used to determine the expression levels of antioxidant oxidase genes *NtCAT1*, *NtPOD1,* and *NtSOD1*; osmoregulation related genes *NtTIP1*, *NtHAK1,* and *NtNXH1*; stress-responsive genes *NtERD10C*, *NtERD10D,* and *NtELA5*; and Hormone synthesis genes *NtNCED3*, *NtABRE*, *NtSNAT1,* and *NtCOMT1*. *NtActin* was used as the endogenous gene. The primers used for qRT-PCR are shown in Supplementary Table S1.

## 5. Conclusions

Overall, our results indicated that LrHSP17.2 is a C-II sHSP that is activated under HS conditions and involved in multiple abiotic stresses by ROS pathways. In addition, it can protect the synthesis of relevant functional proteins and alleviate stress by responding to exogenous hormones and participating in their synthesis pathways.

## Figures and Tables

**Table 1:** Summary of cis-acting elements that are related to plant stress response.

Element	Core	Function	Number
ABRE	ACGTG	ABA response	1
ARE	AAACCA	Anaerobic-induced regulation	3
CGTCA-motif	CGTCA	MeJA response	2
G-BOX	CACGTT	Light response	1
LTR	CCGAAA	Low temperature response	1
MYB	C/TAACCA	MYB transcription factor binding	9
TC-rich repeats	GTTTTCTTAC	defense and stress response	1
W-BOX	TGAC	WRKY transcription factor binding	10
CCAAT-BOX	CCAAT	related to plant heat tolerance	7

## Data Availability

The original contributions presented in the study are included in the article/Supplementary Material, further inquiries can be directed to the corresponding author/s.

## Supplementary Materials

The following supporting information can be downloaded at: https://www.mdpi.com/article/10.3390/plants13172416/s1, Table S1: Primers used in this study; Figure S1: Effect of ABA and MT treatments on heat resistance of *Lilium regale*.
