# Supplementary material for: LrHSP17.2 Plays an Important Role in Abiotic Stress Responses by Regulating ROS Scavenging and Stress-Related Genes in Lilium regale
Source: Plants (Basel). 2024 Aug 29;13(17):2416. doi: 10.3390/plants13172416 (PMC11396892; doi:10.3390/plants13172416)
Supplement: Supplementary file 1 [file plants-13-02416-s001.zip › Supplementary.pdf]

Table S1 List of primers used in this study.

Prime name	Sequence （5′ to 3′）	Usage
LrHSP17.2-F	GCCATGGATTCCAGGATCAT	Full-length PCR
LrHSP17.2-R	TCAAGCGATCTTGACCTCAA	
LrHSP17.2-F	ATTCCAGGATCATCGCACTT	RT-PCR/qRT-PCR
LrHSP17.2-R	TCGTCCTCCACTTGAACCTT	
LrGAPDH-F	ACTTGGTTTCCACTGATTTCCTCG	
LrGAPDH-R	CTTGCTAATGTGGCGGATGAGAT	
proLrHSP17.2-F	GTGTGAGAGTGTGAGTGTGAAGGGCT	Promoter cloning
proLrHSP17.2-R	TAAATCACACTTGGAACAATCCCTAT	
NtCAT1-F	TGGATCTCATACTGGTCTCA	qRT-PCR
NtCAT1-R	TTCCATTGTTTCAGTCATTCA	
NtPOD1-F	CTCCATTTCCATGACTGCTTTG	
NtPOD1-R	GTTGGGTGGTGAGGTCTTT	
NtSOD1-F	GACGGACCTTAGCAACAGG	
NtSOD1-R	CTGTAAGTAGTATGCATGTTC	
NtTIP1-F	ATCTTCGTTTTCGCAGGTCA	
NtTIP1-R	GACAATGTAGAGAATGCCACG	
NtHAK1-F	ATCCACACCGAGCTTGTTTCAGGA	
NtHAK1-R	TGGGTCCAATTCTTCCCACCAAGA	
NtNXH1-F	GAGAACCGCTGGATAAATGAG	
NtNXH1-R	CTTCACTGAAGACCAGAACG	
NtERD10C-F	ACGGACGAATACGGCAATC	
NtERD10C-R	TCTCCTTAATCTTCTCCTTCATCC	
NtERD10D-F	GAGGACACGGCTGTACCAGT	
NtERD10D-R	GCGCCACTTCCTCTGTCTT	
NtLEA5-F	TTGTTAGCAGGCGTGGGTAT	
NtLEA5-R	CTCTCGCTCTTGTTGGGTTC	
NtABRE-F	CTGTCACGGGACATGATTGC	
NtABRE-R	CTAGAGTGTACGCCTGCTTCC	
NtNCED3-F	GCCATCTTCAAGAGACTTGGCTTAT	
NtNCED3-R	AATTGGAGGAGCTTGGAGTGAG	
NtSNAT1-F	GACTCAACCAGATGGAACAGTCG	
NtSNAT1-R	GCTTCTTTTCTCCGTTCCCCT	
NtCOMT1-F	ACTTCCAGAGGCCCCAGATAC	
NtCOMT1-R	CCTTGCGGAAACCAGTAAAAC	
NtActin-F	TGGCATCACACTTTCTACAA	
NtActin-R	CAACGGAATCTCTCAGCTCC	

Fig S1

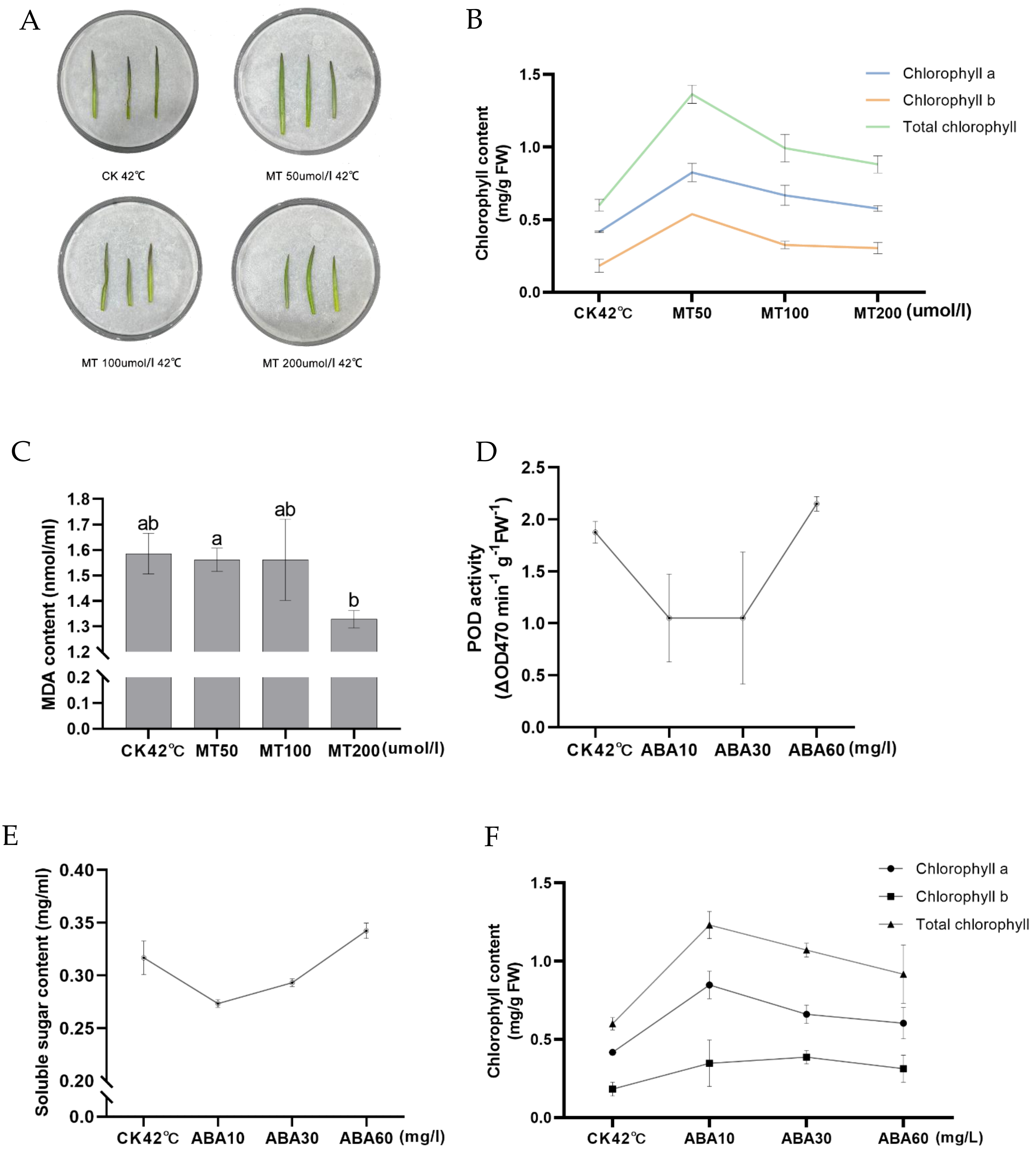

Figure S1. Effect of ABA and MT treatments on heat resistance of *Lilium regale*. (**A**) Phenotypes of CK and MT treatments under heat stress; (**B**) Chlorophyll content of CK and MT treatments under heat stress; (**C**) MDA content of CK and MT treatments under heat stress; (**D**) POD activity of CK and ABA treatments under heat stress; (**E**) Soluble sugar content of CK and ABA treatments under heat stress; (**F**) Chlorophyll content of CK and ABA treatments under heat stress. *t*-test analysis of variance was employed to identify treatment means that differed statistically. Samples with different letters are significantly different: * $p < 0.05$, ** $p < 0.01$, Unmarked means non-significance.
